# Habitat use of urban-nesting lesser black-backed gulls during the breeding season

**DOI:** 10.1038/s41598-019-46890-6

**Published:** 2019-07-19

**Authors:** Anouk Spelt, Cara Williamson, Judy Shamoun-Baranes, Emily Shepard, Peter Rock, Shane Windsor

**Affiliations:** 1 Department of Aerospace Engineering, University of Bristol, Bristol, BS8 1TR United Kingdom; 2 Institute for Biodiversity and Ecosystem Dynamics, University of Amsterdam, Amsterdam, 1098XH The Netherlands; 3 Department of Biosciences, Swansea University, Swansea, SA2 8PP United Kingdom

**Keywords:** Behavioural ecology, Behavioural ecology, Behavioural ecology, Urban ecology, Urban ecology

## Abstract

Increasing urbanisation is detrimental for some animal species and potentially advantageous for others. Urban-nesting populations of gulls have undergone rapid population increases worldwide, which has resulted in an increase in human-gull conflicts. In order to inform management and conservation decisions in relation to these populations, more information is needed about the behaviour of these birds in urban settings and how they utilise their environment. This study combined Global Positioning System (GPS) tracking data of 12 urban-nesting lesser black-backed gulls, *Larus fuscus*, with habitat and behaviour data over three breeding seasons (2016–2018). Despite the proximity of marine areas (~10 km), the birds only made significant use of terrestrial environments, spending two-thirds of their time away from the nest in suburban and urban areas, and one-third in rural green areas. The gulls utilised suburban and urban areas more as their chicks grew and appeared to use diverse foraging strategies to suit different habitats. These results indicate that the range of potential foraging areas available needs to be considered in management decisions and that urban bird populations may not use the resources they are expected to.

## Introduction

Urbanisation of the landscape affects animal populations worldwide and often results in lower species diversity and richness^1,2^. However, some animals can take advantage of urban environments, including various species of gulls, which can use suburban and urban areas for nesting sites and foraging^3^. Gulls traditionally exploit islands or coastal areas for breeding, but across Europe a number of gull species such as lesser black-backed gulls, *Larus fuscus*, herring gulls, *Larus argentatus*, yellow-legged gulls, *Larus michahellis*, and black-legged kittiwakes, *Rissa tridactyla*, now have substantial urban-nesting populations^4–6^. In the United Kingdom (UK), urban gull populations have seen a rapid increase from the mid-1980’s onwards, while non-urban populations have experienced declines over the same period^7,8^. However, national population trends differ per colony with both increasing and decreasing trends in UK colonies of three gull species; herring gulls, lesser black-backed gulls and greater black-backed gulls, *Larus marinus*^9^. A number of possible advantages of nesting in the urban environment have been put forward in relation to the increase in numbers nationally, including warmer temperatures, ample nesting sites, lower predation rates and access to reliable food resources^5^.

Cities are landscapes made up of different habitat types (e.g. buildings, gardens, streets, waste centres) and associated resources within them. Little is known about how gulls nesting in these areas utilise these habitats, or indeed if they only use urban areas for nesting. Bird-mounted GPS based tracking units are an ideal method for measuring movement patterns in detail and have been used to study gulls across Europe^10–14^. However, to date these studies have mainly focused on gulls nesting outside the urban environment and only two published studies (to our knowledge) tracked urban-nesting gulls with GPS devices. A short-term tracking study (<48 h) of ring-billed gulls, *Larus delawarensis*, nesting on the ground on a small island within the city of Montreal, found that the birds preferred to forage in agricultural lands^15^. A one-year study of four herring gulls nesting on roofs in the small coastal town of St. Ives, UK found that the gulls had highly variable individual home-range sizes and activity patterns, and that the birds spent a considerable amount of time away from suburban and urban areas, visiting both marine and agricultural habitats^16^. As such, long-term detailed studies of habitat use by urban-nesting gulls in any substantial urban environment are currently lacking.

The increase of urban gull populations is linked to an increase in conflicts with people, resulting in perceived problems such as aggression, mess, noise, damage to buildings, transmission of diseases and hazards to aircrafts^5,17^. A range of different non-lethal and lethal control measures have been proposed to control urban gull populations such as removal of access to food resources, frightening devices, netting over roof tops, removing nests and egg oiling^5,17^. Although some of these are effective locally and temporarily, they are not on a larger scale^17^. Indeed, the potential effectiveness of large-scale control measures such as removal of access to food resources is difficult to estimate as little is known about the behaviour and habitat use of urban-nesting gulls. Therefore, there is a need to understand the behaviour of these birds in urban settings and how they make use of their environment in order to inform management and conservation decisions in relation to increasing urban gull populations.

The aim of this study was to quantify in detail how urban-nesting gulls utilise their environment and if this changes with breeding stage. This was addressed by specific assessment of: (1) the effect of breeding stage on the habitat use of urban-nesting gulls and (2) the effect of habitat and breeding stage on their time-activity budgets. Based on previous studies^10,18,19^, we hypothesised that the urban-nesting gulls in Bristol would mostly use terrestrial resources, noting however that due to the proximity of the sea (~10 km), the marine environment could still be utilised. We also expected systematic changes in habitat use and time-activity budgets relating to the breeding stage of the gulls based on dietary and foraging behavioural changes observed in previous studies^10,20–23^. Our study focused on urban-nesting lesser black-backed gulls in the city of Bristol, UK. This species is amber listed in the UK and their overall population in the UK decreased by 48% from approximately 91,300 to 43,824 apparently occupied nests (AON) between 2000 and 2013^7,9^. We fitted twelve individuals with long term GPS tracking units^24^ and collected high resolution positional and acceleration data over three breeding seasons (2016–2018). The tracking data were then combined with behavioural data, breeding status and habitat data to quantify the habitat use and time-activity budgets of these urban-nesting gulls.

## Results

### Habitat use

**Figure 1:**
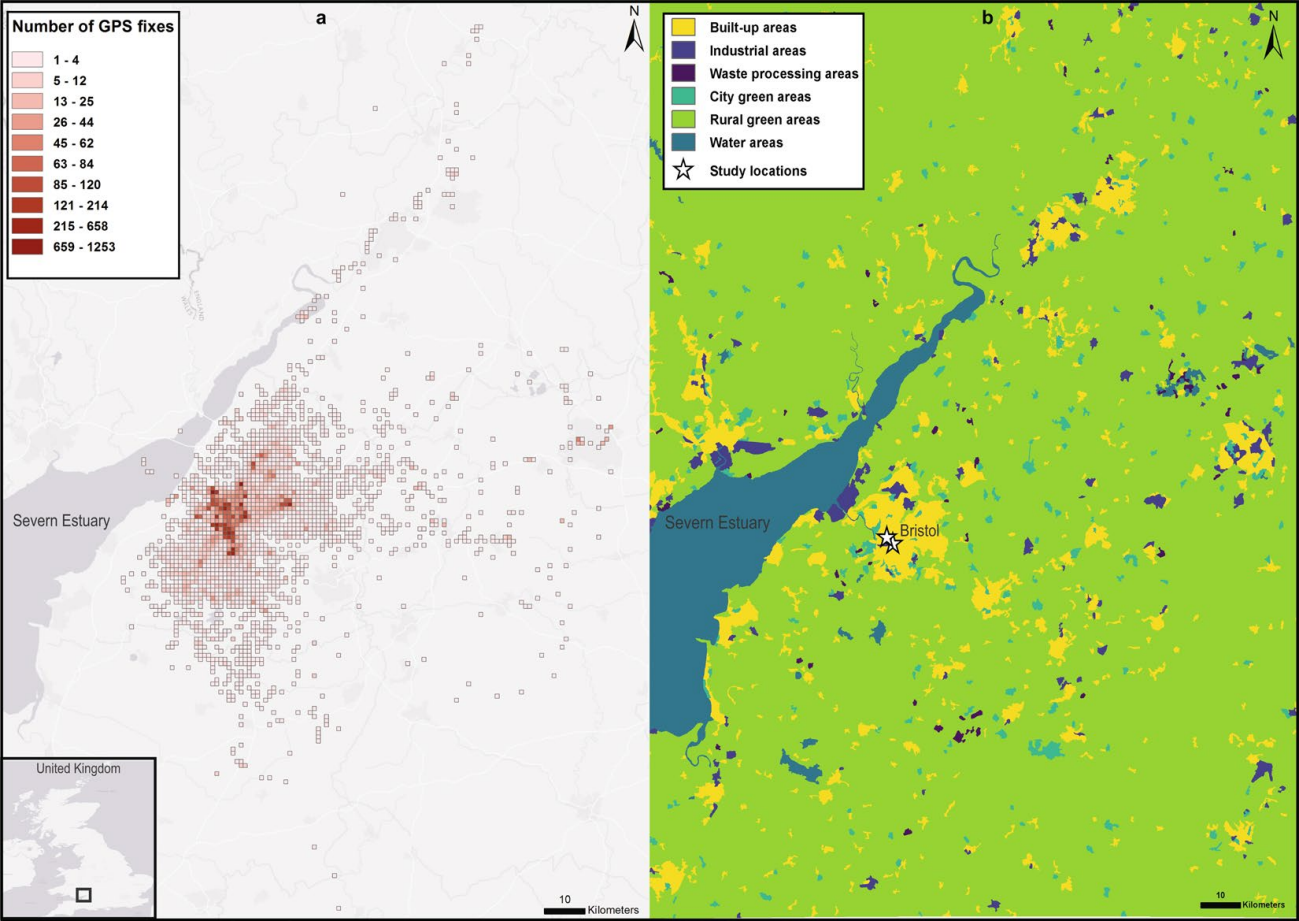
Maps of the density of GPS location fixes in relation to habitat type. (**a**) The number of GPS fixes (filtered to 30 mins) of all individuals during three breeding seasons (2016–2018). Grid cell size was set to 1000 m. Base map sources: Esri, DeLorme, HERE Technologies, MapmyIndia. (**b**) Map of Bristol in the UK coloured by habitat type (Supplementary Table S2). The locations of the two study locations used in this study are marked with a white star (coordinates in decimal degrees for Arts and Social Sciences Library (ASSL): 51.459600, −2.601648 and for dBs music centre (dBs): 51.451582, −2.588388).

**Figure 2:**
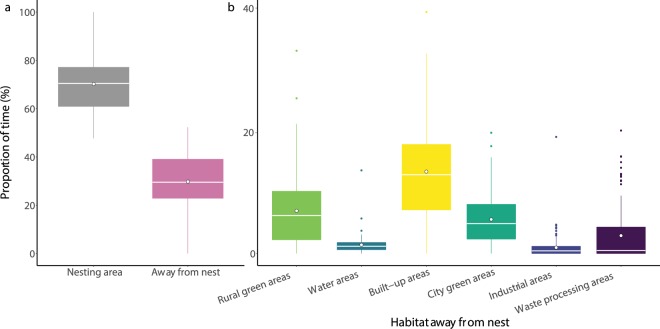
The proportion of time spent during the breeding season. (**a**) Time in the nesting area and time spent away from the nesting area. (**b**) Time in the six different habitat types when away from the nesting area. White dots represent the mean proportion of time.

Our point pattern analysis showed that out of 21,143 GPS fixes away from the nesting area only 5 were in the marine environment (Fig. 1a). These GPS fixes correspond to one individual performing one short trip to the Severn Estuary during the breeding season in 2018. The overwhelming majority of GPS fixes were located on land and were concentrated around the Bristol City area, with up to 1,253 GPS fixes per km^2^, with fixes being taken every 30 minutes. Over the course of the breeding season the gulls spent 29.8 + 2.3% (mean + s.e.m.) of their time away from the nesting area (Fig. 2a) which was defined as a buffer of 50 m around each nest (Supplementary Fig. S1). The birds spent the greatest proportion of this time away from the nesting area in suburban and urban areas (23.2 + 0.4%), which included the main habitat categories: built-up areas (buildings, roads and artificially surfaced areas), city green areas, industrial areas and waste processing areas (Fig. 2b). The gulls also spent a substantial proportion of their time in rural green areas (7.1 + 0.6%), with this main habitat category being largely made up of visits to agricultural fields.

**Figure 3:**
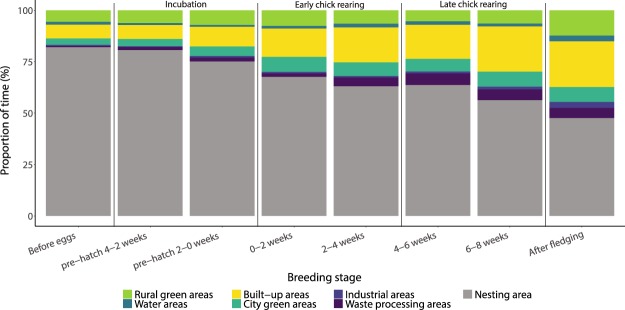
Mean proportion of time spent in the seven different habitat types depending on breeding stage. Time periods of incubation, early chick rearing, and late chick rearing are indicated at the top of the graph. Nesting area was defined as a radius of 50 m for all nests (Supplementary Fig. S1).

The proportion of the total time (including at the nest) spent in specific habitats varied substantially with the stage of the breeding season (Fig. 3). As the breeding season progressed, birds spent less time on the nest and more time overall and proportionally in suburban and urban areas, with the proportion of time spent in suburban and urban areas increasing from incubation (14.5 + 0.7%) to early chick rearing (26.1 + 0.8%) to late chick rearing (32.6 + 1.3%). This increase could mainly be attributed to an increase in the amount of time spent in the main habitat categories built-up areas (incubation: 8.2 + 1.1%, early chick rearing: 15.4 + 1.1, and late chick rearing: 19.3 + 1.3%) and waste processing areas (incubation: 1.6 + 0.6%, early chick rearing: 3.0 + 0.9%, and late chick rearing: 5.5 + 2.2%). Over the same period the proportion of time spent in rural green areas (mainly agricultural fields) remained relatively constant from incubation (6.6 + 1.3%) to early chick rearing (7.0 + 1.1%), to late chick rearing (5.9 + 1.1%). The best model predicting the proportion of time spent included habitat (χ^2^_6_ = 67, p < 0.001), the interaction term habitat * breeding stage (χ^2^_49_ = 2,156, p < 0.001) and random slope of individual (χ^2^_28_ = 2,782, p < 0.001). Therefore, habitat type and breeding stage were important drivers for the proportion of time spent in the habitats, but this proportion differed between individuals (Supplementary Fig. S2).

### Time-activity budgets

**Figure 4:**
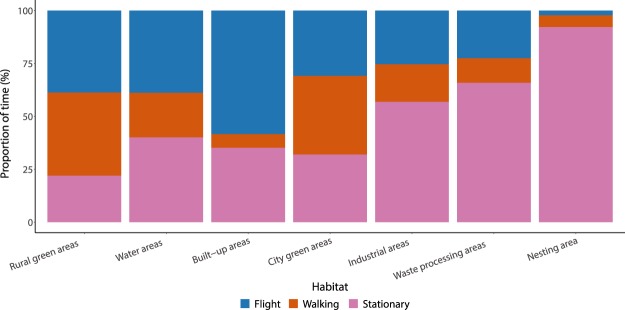
Mean proportion of time spent on each of the three behaviour types in the seven different main habitats. Behaviour classification is based on accelerometer data.

**Figure 5:**
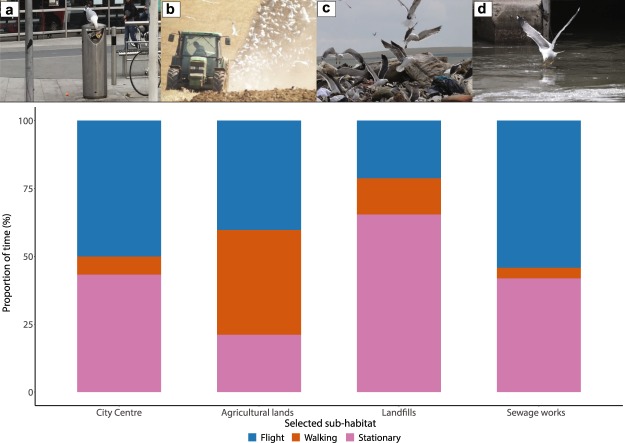
Photographs of gull behaviour taken during observations at four specific feeding grounds within the main habitats and the mean proportion of time spent on each behaviour in those habitats based on accelerometer data. (**a**) Bristol city centre. (**b**) Agricultural lands. (**c**) Landfills. (**d**) Bristol Sewage Works. These specific feeding grounds were selected from the main habitat types: built-up areas (**a**), rural green areas (**b**) and waste processing areas (**c**,**d**).

The accelerometer-based time-activity budgets showed that the gulls spent in general 14.4 + 1.1% of time in flight, 10.4 + 3.0% walking and 75.2 + 1.6% stationary, although time-activity budgets differed between individuals (Supplementary Fig. 3). This proportion of time spent performing different behaviours varied with habitat (Fig. 4). The time-activity budgets in built-up areas and the nesting area were very different from any of the other main habitat types, whereas similar behaviour patterns were seen between rural green and city green areas, and between waste processing and industrial areas. In order to compare the behavioural patterns and the different foraging strategies of gulls, we have highlighted four examples of the time-activity budgets in four specific feeding grounds within the different main habitats (Fig. 5); (1) Bristol city centre (within main habitat built-up areas), (2) Agricultural lands (within main habitat rural green areas), (3) Landfills (within the main habitat waste processing areas), and (4) Bristol Sewage Works (within the main habitat waste processing areas). Time-activity budgets in Bristol city centre and at the Bristol Sewage Works seem to be quite similar, with high proportions of time spent in flight or stationary, and a low proportion of time spent walking. By contrast, on agricultural lands, time-activity budgets showed that gulls spent the largest proportion of their time walking, while in landfills the majority of the gulls’ time was spent sitting or standing.

**Figure 6:**
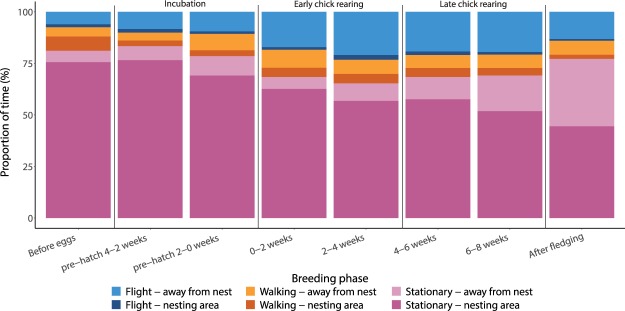
Mean proportion of time spent on each of the three behaviour types either in the nesting area (darker shades) or when away from the nest (lighter shades) depending on breeding stage. Behaviour classification is based on accelerometer data.

The proportion of time spent on different behaviours also varied with breeding stage, resulting in proportionally more time spent stationary and less time spent in flight at the beginning and end of the breeding season (Fig. 6). Meanwhile, the proportion of time spent walking remained similar over the breeding season. The time-activity budgets showed different behaviour patterns in each habitat as the breeding season progressed, but all except waste processing areas showed an increase in stationary behaviour at the end of the breeding season (Supplementary Fig. 4). The best model predicting the proportion of time spent on a behaviour (time-activity budgets) included behaviour (χ^2^_2_ = 46, p < 0.001), the interaction behaviour * habitat (χ^2^_21_ = 852, p < 0.001), behaviour * breeding phase (χ^2^_18_ = 65,869, p < 0.001), the three-way interaction behaviour * habitat * breeding phase (χ^2^_126_ = 2,193, p < 0.001), and random slope of individual (χ^2^_6_ = 259, p < 0.001). This indicates that time-activity budgets varied per breeding stage, per habitat and that the different habitats had distinct changes in activity patterns as the breeding season progressed.

## Discussion

Our study showed that despite the close proximity to the coast (~10 km), the gulls tracked in this study did not make use of the marine environment during the breeding season, except for a single trip by one gull. The degree to which gull colonies use the marine environment is likely to reflect a balance between costs and benefits of different foraging strategies. The birds’ ability to fly the distance to the coast does not appear to be a limiting factor, as the maximum distance away from the colony during the breeding season was 87 km. Other tracking studies with the same species breeding in non-urban areas found maximum foraging ranges from 80^11^ up to 359 km^25^ during the breeding season, with mean foraging ranges of 20–30 km. Gulls nesting on two islands in the Bristol Channel (Steep Holm and Flat Holm), both within foraging range of Bristol (~40 km), have been observed to feed their chicks with marine invertebrates^26^ indicating that the marine area close to Bristol does offer potential food sources. In addition, some of the birds in this study visited the marine areas close to Bristol both before and after the breeding season, indicating that they were aware of this resource but did not make use of it during the breeding season. Although studies with seabirds have shown that a shift to marine resources can be very beneficial during chick-rearing due to high nutrimental value of these resources^20,27^, the gulls in Bristol were selecting to use terrestrial foraging sites over marine foraging areas during the whole breeding season. This suggests that the net energy gain of foraging in the available terrestrial environment is higher than for the local marine environment for these urban-nesting birds and this might reflect the state of the resource availability and foraging costs in the surrounding ecosystem.

The birds in this study appear to forage both in suburban and urban environments, as well as in the rural green areas (mainly agricultural lands) around the city of Bristol. When away from their nest the birds spent on average two-thirds of their time in the suburban and urban areas and one-third of their time in rural green areas such as agricultural fields. When in the suburban and urban areas, it appears likely that many of the gulls in this study would have obtained a substantial amount of food for themselves and their chicks from anthropogenic waste based on the locations they visited, and the behaviours seen in those locations. Indeed, dietary studies have shown that anthropogenic waste can be a large part of the diet of urban-nesting gulls^23,28^. Interestingly on average the birds spent nearly one third of their time away from their nests in the rural green areas around the city. Presumably the gulls were using these areas for foraging as they are often rich in earthworms and insects and these are easier to find in short vegetation or after fields have been disturbed by activities such as ploughing^28,29^. Our personal observations confirmed that gulls were often present when farmers were working on fields and our movement data showed gulls returning to specific fields in the days after they were ploughed. Other studies with large gulls have shown that individuals forage and feed at agricultural lands^12,28^ with a study showing the most common items of food in pellets were coming from this habitat^11^.

We observed a clear decreasing pattern in the proportion of time spent at the nest as the breeding season progressed, with the proportion of time spent in suburban and urban areas (especially built-up and waste processing areas) increasing from incubation to early and late chick rearing. The trend of decreasing time at the nest was expected based on similar patterns in nest attendance observed in non-urban colonies of the same species^10^. The increase in time in suburban and urban areas suggests that resources in these areas, such as human refuse, provide important resources for chick rearing. This is supported by other studies of large gulls showing increased use of suburban areas, city parks^30^ and landfills^22^ from incubation through post fledging. However, results of studies on dietary switching in gulls are mixed as to changes in the proportion of anthropogenic food intake over the breeding season. One study with herring gulls in the UK found a decrease in the proportion of agricultural food and an increase in proportion of anthropogenic waste food^23^, but other large gull studies showed no change in dietary proportions^31–33^, while still others found a decrease in the proportion of anthropogenic food^20,21^. However, trends in dietary studies are difficult to compare with patterns in habitat use measured in GPS tracking studies due to the different limitations of the methods. Diet analysis methods do not provide information about where the food has been obtained by the individual and often underestimate the amount of soft and fully digestible food, such as bread^34^. GPS tracking studies are able to indicate where the food has been obtained but cannot provide information as to the type and amount of food obtained in the areas visited.

We propose three - not mutually exclusive – hypotheses to explain the increase in the proportion of time spent in suburban and urban areas from incubation to chick rearing as the chicks’ food demand increases. (1) Suburban and urban food resources are readily available and more predictable in space and time than rural food sources^1,35^. Human activities, such as daily feeding of birds in gardens, weekly waste collection from the streets and daily operating waste processing centres, are providing gulls with a predictable and widely available food resource. On the other hand food resources from rural green areas, such as earthworms and insects, are present when the soil is disturbed by ploughing on the land, which takes place at irregular times, and when local weather conditions increase arthropod availability, e.g. damp or wet ground^28,29,36^. (2) Suburban and urban food resources have a higher energetic value than rural food resources. The energetic content of waste has been calculated to be 2.2 calories/gram, whereas for earthworms this is only 0.71 calories/gram^33^. With observed ingestion coefficients (rate of increase of mass) of herring gulls^37^, the net rate of energy intake would be higher during feeding on waste (28 calories/hour) than on earthworms (23 calories/hour). (3) Suburban and urban food resources are closer to the nesting area. A shorter distance from the nest would imply less commuting time and therefore possibly shorter, more frequent, and more efficient foraging trips with a higher net energy intake. For example, a study on lesser black-backed gulls in a traditional island colony showed that the foraging trip duration was shorter during chick rearing in comparison to during incubation when parents only have to feed themselves^10^. Currently, data are not available to test all three hypotheses, therefore this study is not able to differentiate between them, with the possibility that all three play a role.

The gulls had distinct time-activity budgets associated with each habitat type, which appeared to reflect the use of different foraging strategies in four specific feeding grounds (Fig. 5). At waste processing areas, such as landfills, the main behaviour observed was “stationary” behaviour. Together with our personal observations this suggests a “sit-and-wait” strategy, where the birds would wait until new waste was unloaded before flying in and feeding. A particularly characteristic behaviour was observed at the Bristol Sewage Works, where gulls would wait lined up along the wall of the sewage flow and then fly down to snatch food waste from the sewage water that flowed past. This behaviour was confirmed by the time-activity budget (Fig. 5d) where the proportion of time spent in flight was larger than in landfills. On agricultural lands, time-activity budgets showed that gulls spent the largest proportion of their time walking. This strategy is frequently used by gulls to feed on both invertebrates and insects in fields^26^. Lastly, in built-up areas, such as the city centre, besides the ‘sit-and-wait’ approach, the main strategy seems to be flying and actively searching for feeding opportunities from the air. Overall these different time-activity budgets related to habitat type probably reflect the availability of resources and the foraging strategies needed to acquire them in each of the habitat types, with different costs and intake resulting in differing profitability for each habitat.

The change in the birds’ time-activity budgets over the breeding season suggests that they shift some of their resting behaviour to their foraging grounds. The overall proportion of stationary behaviour decreased from incubation – when the vast majority of stationary behaviour occurs at the nest – to chick rearing. However, during chick rearing, while the overall time spent stationary stays relatively constant, a progressively greater proportion of stationary behaviour occurs away from the nest. This shift in stationary behaviour to other habitats away from the nest may allow them to forage more efficiently. Nest attendance is assumed to be important during incubation and early chick rearing stages when either the clutch or chicks need to be protected^38^. Then during later chick rearing stages returning to the nest to rest and protect the chicks may become less important, as the chicks are now better able to fend for themselves. Indeed, gulls have been observed resting away from the colony during the breeding season^19,39^ and resting at feeding grounds might be energetically more efficient than flying back to the nest. Moreover, an increase in stationary behaviour away from the nest could indicate an increase of the “sit-and-wait” foraging strategy which may be less energetically demanding than flying and actively searching for food. At the end of the breeding season (~8 weeks), the time spent stationary increased again, which could be related to the fledging chicks being able to leave the nest. Chicks have been seen leaving the nest with their parents and being fed by them at different feeding grounds (pers. obs). These results suggest that these birds may shift their time-activity budgets during the breeding season to be able to rest at the foraging grounds and therefore gain energy more efficiently.

In the UK several gull species are amber listed, with varying rates of population decline^7^. Gull populations in cities however are increasing which is resulting in higher numbers of human-gull conflicts. With respect to these trends in urban gull populations, a range of lethal and non-lethal control measures are currently being implemented^3,5^. When considering these measures for urban-nesting gulls it would appear that although suburban and urban areas may provide the majority of foraging opportunities during the breeding season, that the birds are also foraging outside of these areas and are capable of utilising a wide range of food sources. This needs to be taken into account when applying control measures, such as the removal of access to waste processing centres, as the birds may easily shift their foraging efforts to other food sources. This shifting of foraging effort with changes in the availability of point food sources has been documented in gulls^40,41^ and other bird species^42–44^. In addition, it is clear from this study that the birds ranged over a large proportion of the suburban and urban environment and that the individuals seen in a particular location could be nesting in a distant part of the city. Other studies with gulls show that non-urban nesting individuals may also forage in urban areas well away from their colony^5,24,30^. Together this indicates that local control measures for nesting birds may not have an effect on the number of birds in that area. Human-wildlife conflicts are not only observed in gulls, with other species that thrive in urban environments being reported to cause problems with damage to properties, disease transmission, and aggression^45^. Understanding the behaviour and habitat use of urban-living animals is therefore crucial when trying to control and/or prevent conflicts between people and wildlife in cities.

Overall these results show that these urban-nesting gulls spent the majority of their time in suburban and urban areas during the breeding season, while also utilising rural areas surrounding the city to a reasonable extent. The birds however did not make any use of the marine areas close to the city and this is likely to be due to the availability of terrestrial environments offering more efficient foraging opportunities. They appeared to use different foraging strategies to suit different habitats. When considering conservation measures for gull species as a whole, this study supports the view that gulls are generalist opportunistic foragers, taking advantage of a wide variety of food sources^10,11,46,47^. The birds utilised suburban and urban areas more as their chicks grew and their time-activity budgets were variable over time and between habitats. Temporal variability in behaviour and habitat use during the breeding season is also observed in other bird species^48,49^. Overall, this raises the possibility that bird species and populations which might be considered as urban living^1,2^, may make use of resources from outside urban areas and that their behaviour and habitat use may change over the course of the breeding season. This potentially needs to be considered when designing conservation measures for any urban species.

## Methods

### Study area and species

This study was carried out in the city of Bristol, UK (Fig. 1b). The city covers an area of approximately 110 km^2^ with a human population of 459,300^50^. The Severn Estuary and the open sea are located ~10 km from the city centre. Lesser black-backed gulls nesting on two buildings in the city centre were tagged in this study (Fig. 1b – white stars); the Arts and Social Science Library (ASSL) at the University of Bristol and the dBs Music building (dBs) in the centre of Bristol. The two study locations are situated approximately 1.5 km from each other (see Supplementary Methods for more details about the study area and species).

### GPS devices and attachment

Breeding adults were caught at their nest during the first or second week of incubation with either a walk-in chicken wire trap or metal trap-door cage. Eggs were replaced with fake eggs to prevent damage and overheating during warm days. The handling time was minimized (mean: 28 min, range: 16–50 min) to prevent high stress levels and irreversible changes in behaviour. In 2016, 5 birds were caught and tagged with UvA-BiTS GPS devices^24^ at ASSL. In 2017 an additional 7 birds were caught and tagged, one at ASSL and 6 at dBS which resulted in a total of 12 individuals. Unfortunately, 1 GPS device (Individual 1) stopped working after a week therefore this individual has been excluded from this study.

The GPS devices were attached using a wing harness made from tubular Teflon™ ribbon (Bally Ribbon Mills 8476-0.25”). The wing harness method has been found to be the best method of attaching a GPS device for gulls^51^. The mass of both unit and harness was 18 g, which was <3% of the birds’ body mass (mean: 2.4, range: 2.1–2.7). Mass of the birds was quantified by weighing the individuals in a bag attached to an electronic scale (1 g precision) and sex was determined by comparing the size and depth of the bill to wing length^52^. The 5 individuals caught in 2016 were all females and from the 7 birds caught in 2017, 2 were males. All individuals were colour ringed. Supplementary Table S1 provides detailed information about each individual gull. The UvA-BiTS GPS devices are lightweight, solar powered units with rechargeable batteries, and have tri-axial accelerometers and temperature sensors. They log on-board and the data can then be accessed remotely via a Zigbee two-way radio transceiver. The tri-axial accelerometer measures linear acceleration in three directions; X (surge), Y (sway) and Z (heave). Data was downloaded to a field laptop regularly via the radio transceivers placed at the study locations.

### Monitoring breeding stage and device effects

Monitoring of the nests were conducted with a telescope (Swarovski STX 30–70 x95) from overlooking buildings to determine breeding stage with laying, hatching and fledging dates being recorded where possible (For an overview of breeding parameters see Supplementary Methods). Monitoring was performed weekly in March and August (pre-egg laying and after fledging) and twice a week from April until fledging of the chicks (end July). Monitoring continued until a majority of the nests had been checked. The incubation period was defined as from when the first egg was laid until the first egg had hatched (generally four weeks), and the chick rearing period was defined as from when the first egg had hatched until 8 weeks later (generally the fledging age of chicks) or until the chicks had died. These breeding periods were determined separately per individual per year. The GPS data was assigned to specific breeding stages which were defined in two-week intervals for more detailed analysis.

Attaching GPS devices and other transmitters to free-living birds can have negative effects on their behaviour and survival^53^. Previous studies using the same GPS device and harness as in the current study have observed no short- or long-term effects on lesser black-backed gulls^25,54,55^. To test for tag effects on breeding output, we compared the breeding success of our tagged individuals with control gulls nesting on the same roof or adjacent roofs in order to identify possible tag effects. For all three years, no difference was found between number of chicks hatched (χ2_1_ = 0.002, p = 0.961) and number of chicks fledged (χ2_1_ = 2.4, p = 0.124). All work was approved by the University of Bristol Animal Welfare and Ethical Review Body (UIN UB/15/069). Bird handling, tagging and temporary egg removal was conducted under BTO permit A/2831. All work was carried out in accordance with the relevant guidelines and regulations.

### Habitat map

A habitat map was created to assign each GPS location to a habitat type in ArcGIS^56^. This map was based on the 2 m resolution Corine Land Cover European seamless vector database^57^. Several layers with similar spatial resolution were added to the map to improve local habitat types. These layers include data from a landfill database^58^, allotment database^59^, and river and lakes database^60^. Additionally, we added an extra layer of habitat types which included sites that were frequently visited by the gulls and the nesting areas (see below). This resulted in a dataset of 47 different habitat types which were combined to create a dataset with 7 main habitat types: (1) nesting area, (2) rural green areas, (3) water areas, (4) built-up areas, (5) city green areas, (6) industrial areas and (7) waste processing areas (Supplementary Table S2). In this study, the latter four types are collectively referred to as suburban and urban areas. Rural green areas were mainly characterised by agricultural land, forests and meadows. Water areas include rivers, lakes, intertidal areas and the sea.

During the breeding seasons of 2016 and 2017 we inspected sites which were frequently visited by the birds as shown by the GPS tracks in order to create the extra layer of habitat types. These sites included agricultural lands, waste processing centres in and outside of Bristol, and areas such as city parks, sports fields, suburban gardens and schools. These observations were used to assess if the basic habitat map (CLC) identified these locations correctly. If this was not the case, they were added to the layer. During these visits we also noted bird behaviours at specific feeding sites to provide some ecological and behavioural context. These observations were not systematic and are referred to as personal observations.

### Data processing

#### Data preparation

This study focussed only on actively breeding birds, therefore part of the data for three gulls was excluded as they did not breed in the subsequent year (Supplementary Table S1). Additionally, only data within the breeding period was included in this study, e.g. when a nest failed the data collected after this point was removed. This resulted in different number of fixes contributing to each individual’s dataset, however this assured that the habitat use was linked to breeding behaviour and not to behaviour of failed or non-breeders. Additionally, the GPS devices recorded at intervals between 4 and 1,800 seconds during the breeding season and between 1,800 and 3,600 seconds outside the breeding season. Data was filtered to a 30-min rate for habitat use analysis to create equal sampling rates during the breeding season.

#### Habitat use

To demonstrate the distribution of urban-nesting gulls in Bristol, we conducted a point pattern analysis on the filtered 30-min data set of all individuals during the three breeding seasons (2016–2018). Data within the nesting areas were excluded from this analysis based on a cut-off radius of 50 m per nest (Supplementary Fig. S1), resulting in a total of 21,143 GPS fixes used for this analysis. A uniform grid was created with a cell size of 1000 m and the same extent of the GPS fixes. For each grid cell the number of GPS fixes within this grid cell was calculated giving the total number of points per grid cell.

In order to assess how urban-nesting gulls use their surrounding environment we included GPS fixes collected both in flight and on the ground. As we were interested in both general habitat use and foraging behaviour, excluding flight behaviour from the analysis would not be justified. Also, as gulls are opportunistic foragers searching flight cannot definitively be distinguished from commuting flight based on the data collected. Data within the nesting areas were included in this analysis. The filtered 30-min data was used to quantify the habitat use and the effect of breeding stage on habitat use. The response factor was habitat use and was defined as the proportion of time spent in each habitat during a specific breeding stage. The breeding stages were defined per individual per year and set at zero on hatching day. Breeding stage was classified using two-week intervals: before egg laying (pre-hatch 6-4 weeks), pre-hatch 4–2 weeks, pre-hatch 2–0 weeks, 0–2 weeks after hatching, 2–4 weeks, 4–6 weeks, 6–8 weeks and after fledging (8–10 weeks).

#### Time-activity budgets

Acceleration data was collected after each GPS fix at a frequency of 20 Hz for 1 or 2 seconds which means that the acceleration data was coupled to a particular GPS location for that individual. The acceleration data was then used to quantify the behaviours of the gulls using a machine learning classifier created by Shamoun-Baranes *et al*.^61^. That study annotated behaviour of lesser black-backed gulls nesting on an island in the Netherlands based on video data, simultaneous acceleration data and expert knowledge. This annotated dataset plus a set of 14 selected features was used to create a random forest classifier which predicted behaviour. This same classifier was used in this study resulting in the same ten activity classes: “soaring”, “flapping”, “extreme flapping”, “mixed flight”, “walking”, “pecking”, “float”, “boat”, “stationary”, and “other”. Supplementary Table S3 explains these activity classes in detail. For this study, we were mainly interested in three major activity classes: “flying”, “walking” and “stationary”. Therefore, the activity classes “soaring”, “flapping”, “extreme flapping” and “mixed flight” were combined as “flying”. The “pecking” activity class was found to be similar to “walking”, therefore these activity classes were combined as “walking”. Additionally, the activity classes “boat” and “stationery” were similar and reclassified as “stationary”. Lastly, the activity class “float” was reclassified as “other” due to the low sample size of this behaviour. The behavioural data was combined with the GPS locations and the habitat map to compare time-activity budgets between habitats using the proportion of time spent performing each behaviour in each habitat. Data within the nesting areas were included in this analysis.

### Analysis

To analyse the birds’ habitat use and time activity budgets, two generalised linear mixed models (GLMMs) with poisson distribution and logit link were fitted with the lme4 package^62^ in R version 3.5.3^63^. To analyse the bird’s habitat use, the proportion of time spent in each habitat was modelled by adding time spent in each habitat as a response variable and an offset of log(total time spent). Additionally, habitat and an interaction between habitat and breeding phase were included as fixed factors, and a random slope for individual was included to control for within-subject effects (Table 1: model 1). To analyse time-activity budgets in the different habitats and the effect of the breeding stage on these time-activity budgets, the proportion of time spent on each behaviour was modelled by adding time spent on each behaviour as response variable and an offset of log(total time spent). Additionally, a random slope for individual was included. The following fixed factors were included in the model: a) behaviour, b) an interaction term between habitat and behaviour, c) an interaction term between breeding phase and behaviour, and d) a three-way interaction term between breeding phase, habitat and behaviour (Table 1: model 2).

**Table 1:** Model selection of the two models based on likelihood ratio tests.

Model		Fixed terms	Random terms	Test	AIC	AICc	BIC	logLik	deviance	Chisq	df	p
**1**	1a	Habitat + Habitat * Phase			8,465	8,473	8,726	−4,176	8,353			
	1b	Habitat + Habitat * Phase	Habitat/ID	1a vs 1b	5,738	5,759	6,130	−2,785	5,570	2,782	28	<0.001
	1c		Habitat/ID		7,851	7,854	7,987	−3,897	7,793			
	1d	Habitat	Habitat/ID	1c vs 1d	7,769	7,799	7,959	−3,863	7,726	67	6	<0.001
	**1e**	Habitat + Habitat * Phase	**Habitat/ID**	**1d vs 1e**	**5,738**	**5,759**	**6,130**	**−2,785**	**5,570**	**2,156**	**49**	**<0.001**
**2**	2a	Behaviour * Habitat + Behaviour *Phase + Behaviour * Phase * Habitat	Behaviour/ID		12,745	12,772	13,706	−6,205	12,409			
	2b	Behaviour * Habitat + Behaviour *Phase + Behaviour * Phase * Habitat	Behaviour/ID	2a vs 2b	12,498	12,527	13,493	−6,075	12,150	259	6	<0.001
	2c		Behaviour/ID		81,124	81,083	81,164	−40,555	81,110			
	2d	Behaviour	Behaviour/ID		81,083	81,124	81,134	−40,532	81,065	46	2	<0.001
	2e	Behaviour * Habitat	Behaviour/ID	2c vs 2d	80,272	80,273	80,444	−40,106	80,212	852	21	<0.001
	2f	Behaviour * Habitat + Behaviour * Phase	Behaviour/ID	2d vs 2e	14,439	14,441	14,713	−7,172	14,343	65,869	18	<0.001
	**2g**	Behaviour * Habitat + Behaviour *Phase + Behaviour * Phase * Habitat	**Behaviour/ID**	**2e vs 2f**	**12,498**	**12,527**	**13,493**	**−6,075**	**12,150**	**2,193**	**126**	**<0.001**

First the random terms were selected keeping fixed terms the same, and secondly the fixed terms were defined with the best selection of random terms. The model highlighted in bold is the final ‘best’ fit model for that analysis (1e and 2g respectively). Both models resulted in a GLMM model with a random slope. Stage = breeding stage, AIC = Akaike information criterion, AICc = corrected Akaike’s information criterium, BIC = Bayesian information criterion, logLik = log-likelihood ratio statistic, Chisq = Chi-square statistic, df = degrees of freedom. The significance level was set at α = 0.05.

Following Zuur *et al*.^64^ we conducted a multiple step process to select the “best-fit” model. The optimal structure was defined by comparing several information criteria, including the Akaike’s information criterion (AIC), corrected Akaike’s information criterium for small sample sizes (AICc) and Bayesian information criterion (BIC). Additionally, likelihood ratio tests were performed to assess if variables significantly improved the model. The final models can be found in Table 1. Model validation was done by looking for patterns in residual plots and checking heteroscedasticity, uniformity, zero-inflation and overdispersion with the DHARMa package^65^. Overdispersion was assessed by comparing the ratio of actual to expected variance. The significance level was set at α = 0.05 and for results mean and standard error are reported unless stated otherwise.

## Supplementary information

Supplementary Information

Dataset 1

Dataset 2

Dataset 3

## Data Availability

All data generated or analysed during this study are included in this published article (and its Supplementary Information files).
