# Supplementary material for: Habitat use of urban-nesting lesser black-backed gulls during the breeding season
Source: Sci Rep. 2019 Jul 19;9:10527. doi: 10.1038/s41598-019-46890-6 (PMC6642139; doi:10.1038/s41598-019-46890-6)
Supplement: Supplementary file 1 — Supplementary Information [file 41598_2019_46890_MOESM1_ESM.docx]

## **Supplementary Information for:**

Habitat use of urban-nesting lesser black-backed gulls during the breeding season

**Anouk Spelt^1^, Cara Williamson^1^, Judy Shamoun-Baranes^2^, Emily Shepard^3^, Peter Rock^1^, and Shane Windsor ^1^***

**^1^**Department of Aerospace Engineering, University of Bristol, Bristol, BS8 1TR, United Kingdom

**^2^**Institute for Biodiversity and Ecosystem Dynamics, University of Amsterdam, Amsterdam, 1098XH, The Netherlands

**^3^**Department of Biosciences, Swansea University, Swansea, SA2 8PP, United Kingdom

[**^*^**shane.windsor@bristol.ac.uk](mailto:*shane.windsor@bristol.ac.uk)

**Supplementary methods**

*Study area and species – extra information*

Lesser black-backed gulls nesting on two buildings in the city centre were tagged in this study (Figure 1b – white stars); the Arts and Social Science Library (ASSL) at the University of Bristol and the dBs Music building in the centre of Bristol. ASSL is a large building (LxWxH is 41x48x13 m) in the middle of the University of Bristol. Nesting has been recorded since at least 2004 (P. Rock, personal observation) and on average 6 to 8 gulls were nesting on this roof during 2016-2018. Nests were spread out (>20 m between nests) and built upon the stone roof structure. dBs Music is a smaller building (LxWxH is 34x16x11 m) in the centre of Bristol. Nesting has been recorded since 1980 (P. Rock, personal observation) and on average 8 to 10 gulls were nesting on this roof during 2017-2018. Nests were closer to each other on this roof (>5 m between nests) and the stone roof structure contained a mossy layer on which nests were built.

The first large gulls started to populate the roofs of Bristol as early as 1972 ^54^. Between 1998 and 2002 the nationwide survey of seabirds "Seabird 2000" recorded 850 apparently occupied nests (AON) of lesser black-backed gulls in the city ^26^. a gulls species which is amber listed in the UK due the fact that they are considered localised (70-80% of the UK breeding population being located in 10 or fewer Important Bird Areas ^7^).

*Breeding activity*

On average the gulls were tracked for 128±26 days (range: 19-299) during the three breeding seasons (2016-2018, Supplementary Table S1), with five birds being tagged at the beginning of the 2016 breeding season and then an additional seven birds in 2017. Mean first egg laying date was 5th May in 2016 (range: 15/04-02/06), 3th May in 2017 (range: 26/04-22/05) and 10th May in 2018 (range: 30/04-22/05). Mean hatching date of the first egg was 2rd June in 2016 (range: 20/05 - 22/06), 28th May in 2017 (range: 24/05 - 13/06) and 4rd June in 2018 (range: 28/5-13/6). During the breeding season of 2016 all five individuals produced chicks, with three individuals successfully fledging chicks (Supplementary Table S1). In 2017 nine individuals produced eggs, chicks hatched in seven nests and four individuals had one or two chicks successfully fledged. In 2018 eight individuals produced eggs of which chicks hatched in six nests and five individuals had one or two chicks successfully fledged.

**
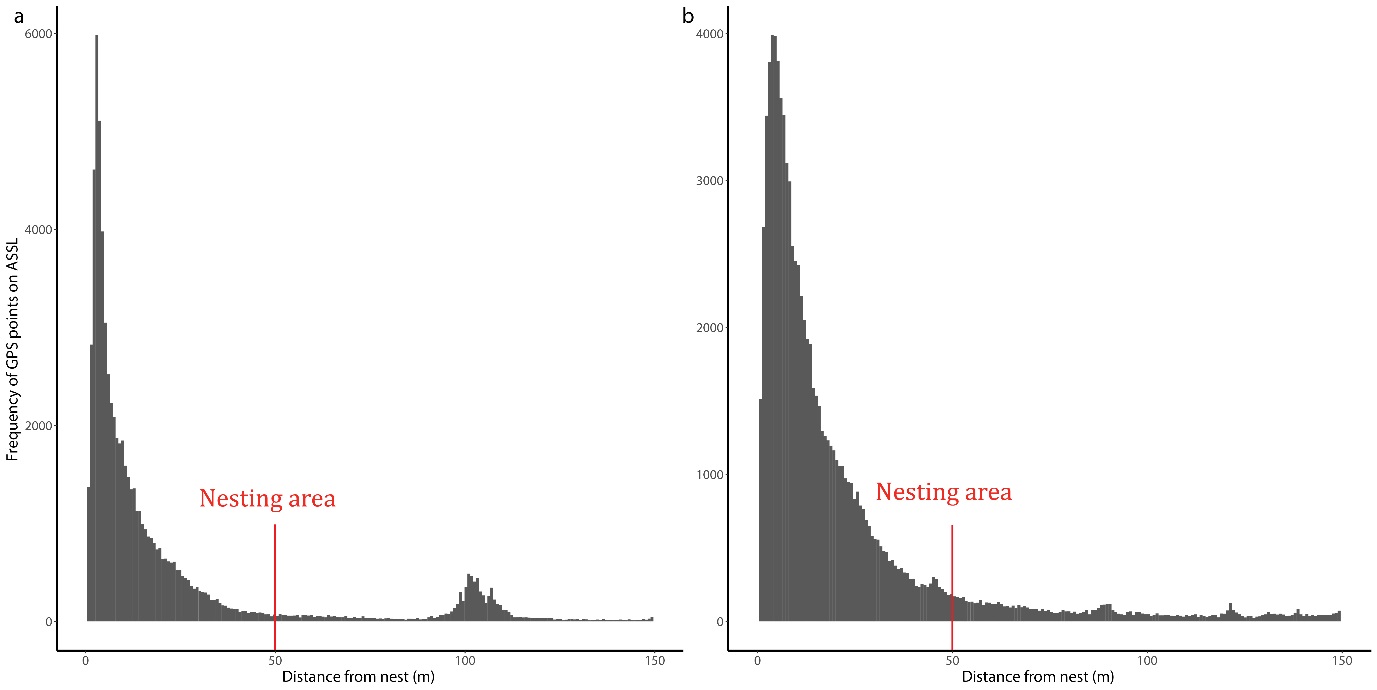
Supplementary Figure S1:** The frequency of distance from each nest (m) per GPS measurement. a) Arts and Social Sciences library (ASSL). b) dBs Music Centre (dBs). The cut-off for defining the nesting area was determined visually and was defined as a radius of 50 m for all nests.

**
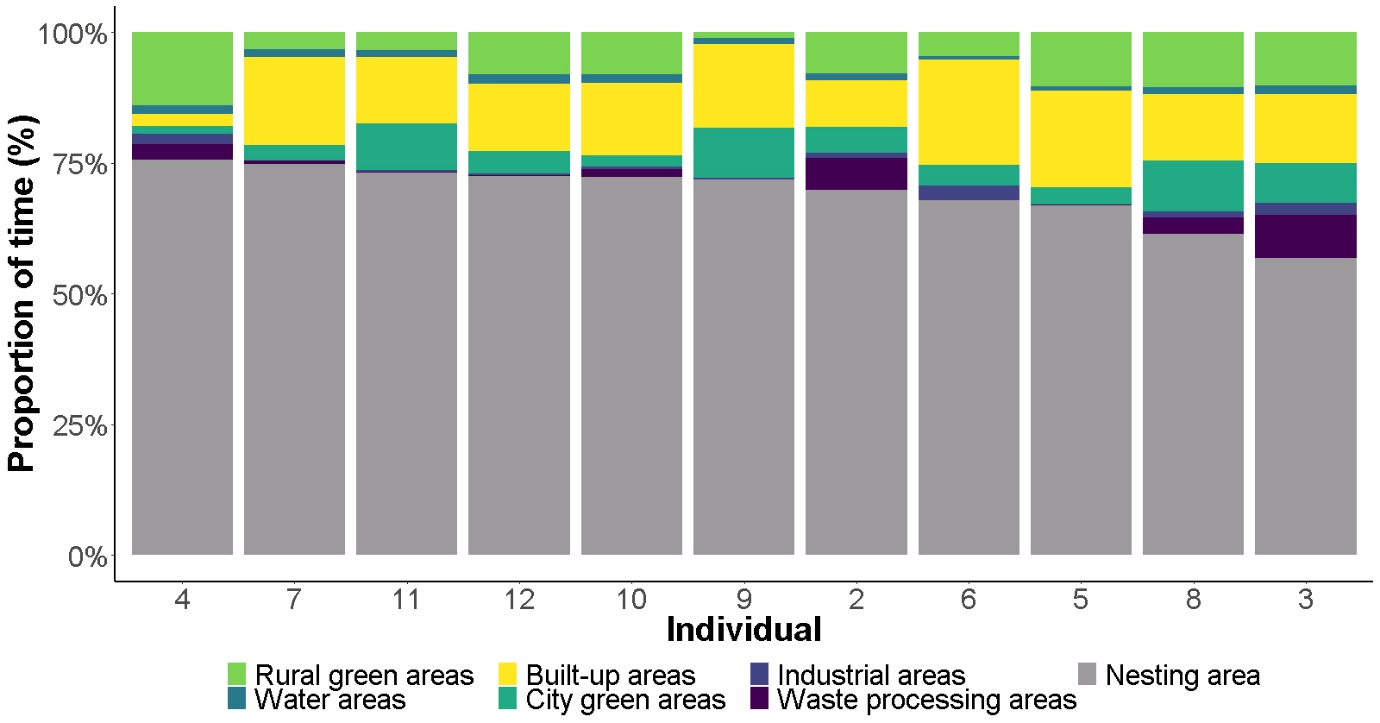
**

**Supplementary Figure S2:** The mean proportion of time spent in seven different habitats by individual urban-nesting gulls in Bristol during three breeding seasons (2016-2018). Individuals are shown from left to right in descending order of proportion of time spent in the nesting area. The data of individual 1 is not included because it was excluded from this study.

**
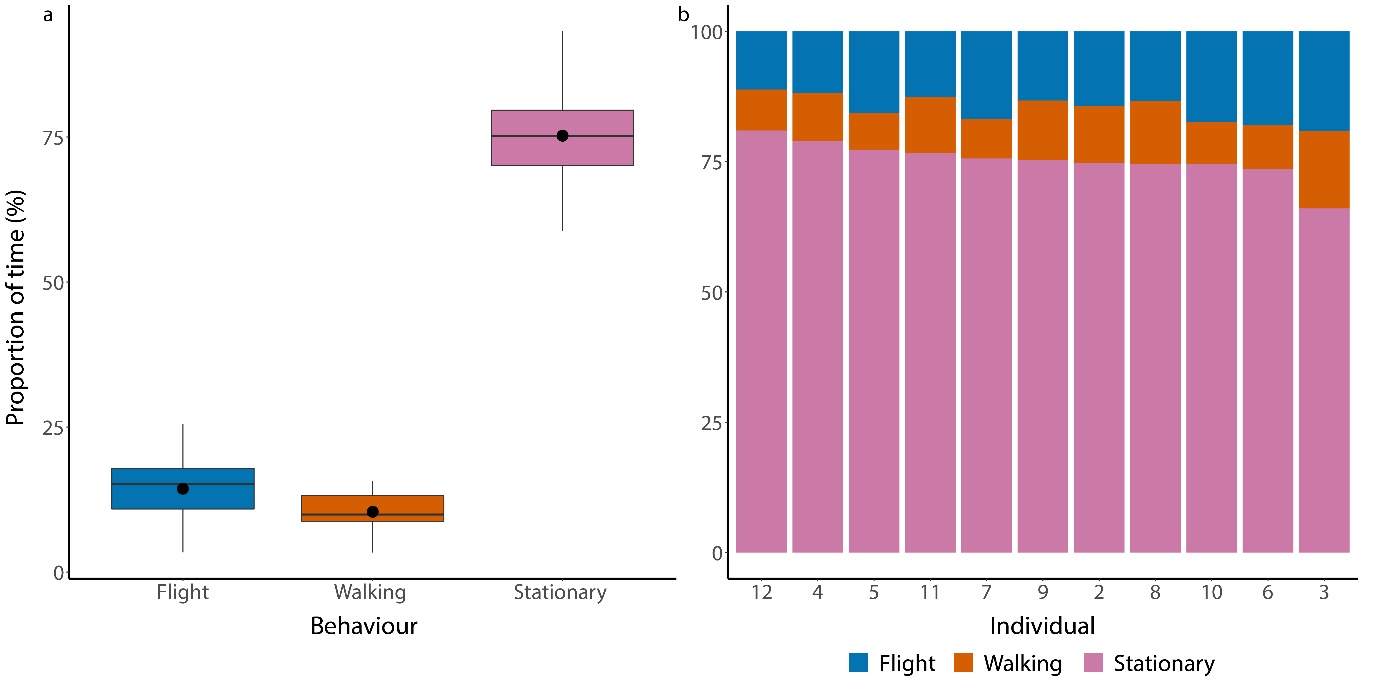
Supplementary Figure S3:** Mean proportion of time spent on each of the three behaviour types during three breeding seasons (2016-2018). a) Mean over the whole breeding season. b) Mean per individual


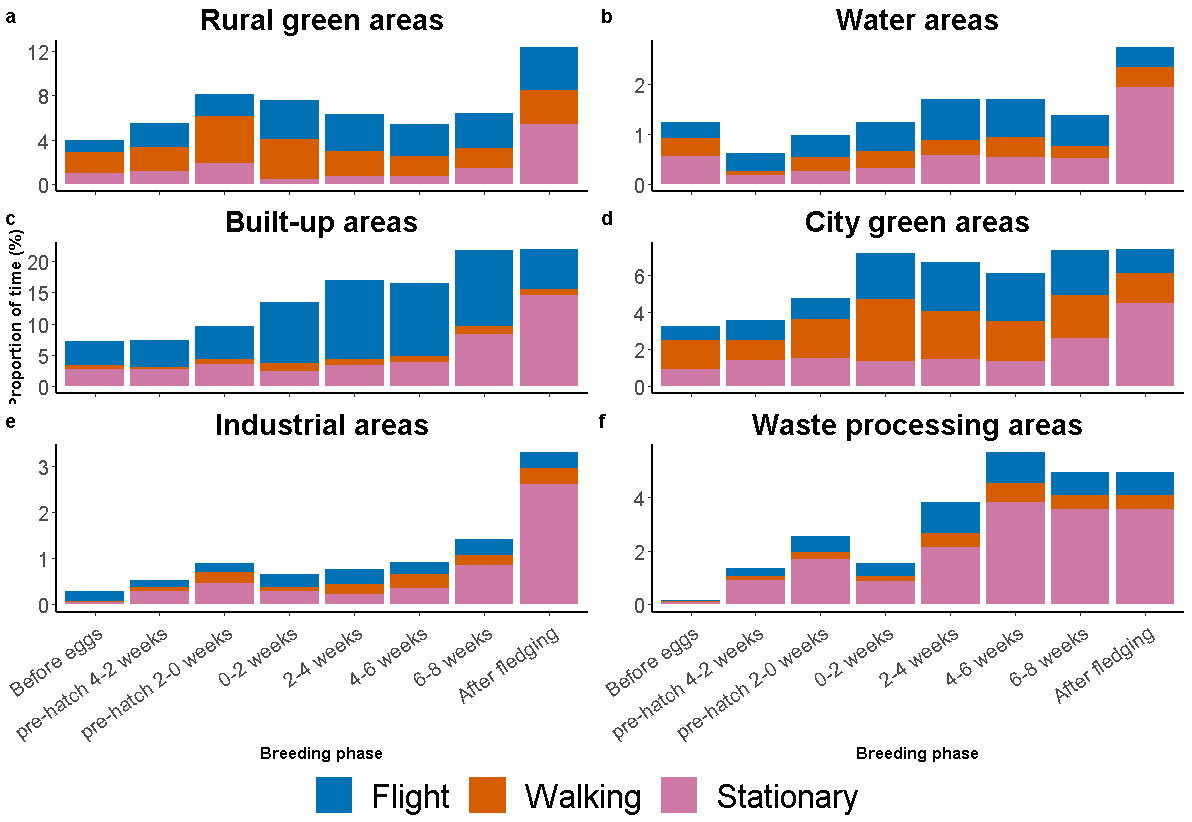


**Supplementary Figure S4.** Mean proportion of time spent on each of the three behaviour types depending on breeding stage for the different habitats when away from the nesting area. a) Rural green areas. b) Water areas. c) Built-up areas. d) City green areas. e) Industrial areas. f) Waste processing areas. g) Nesting area. Behaviour classification is based on accelerometer data

**Supplementary Table S1:** Overview of 12 individual gulls followed during three breeding seasons (2016-2018) in Bristol. In 2016, 5 individuals were tagged, and one GPS device failed after a week (id 1). In 2017, another 7 individuals were tagged, and one GPS device stopped working in 2018 (id 7). The birds were nesting on two different roofs; Art and Social Sciences Library (ASSL) and dBs music Centre (dBs). Days, fixes and fixes per day (fix/day) are given for data when birds were breeding and include the nesting area (30-min data set). Laying dates (lay) and hatching dates (hatch) are given for each individual for each year. Nest info gives information about the state of the nest at the end of each of the breeding seasons; individual was not breeding (no nest), breeding at another colony (other nest), chicks did not hatch (eggs), chicks died during the season (died) or the chicks fledged at the end of the season (fledged).

| **ID** | **Sex** | **Start date** | **End date** | **Roof** | **Breeding only** | | | **2016** | | **2017** | | **2018** | | **Nest info** | | |
| --- | --- | --- | --- | --- | --- | --- | --- | --- | --- | --- | --- | --- | --- | --- | --- | --- |
|  |  |  |  |  | **Days** | **Fixes** | **Fix/day** | **Lay** | **Hatch** | **Lay** | **Hatch** | **Lay** | **Hatch** | **2016 2017** | | **2018** |
| 1 | F | 04/05/2016 | 11/05/2016 | ASSL |  |  |  | 01/May | 30/May |  |  |  |  | Fledged |  |  |
| 2 | F | 04/05/2016 | Ongoing | ASSL | 207 | 9,879 | 48 | 04/May | 27/May | 22/May | 13/Jun | 22/May | 11/Jun | Fledged | Died | Died |
| 3 | F | 04/05/2016 | Ongoing | ASSL | 299 | 14,058 | 47 | 15/Apr | 20/May | 30/Apr | 25/May | 05/May | 28/May | Fledged | Fledged | Fledged |
| 4 | F | 05/05/2016 | Ongoing | ASSL | 33 | 1,537 | 47 | 04/May | 07/Jun |  |  |  |  | Died | No nest | Another nest |
| 5 | F | 09/06/2016 | Ongoing | ASSL | 19 | 888 | 47 | 02/Jun | 22/Jun |  |  |  |  | Died | No nest | No nest |
| 6 | F | 18/05/2017 | Ongoing | ASSL | 72 | 3,449 | 48 |  |  | 10/May |  | 22/May | 13/Jun |  | Eggs | Died |
| 7 | F | 08/05/2017 | 20/05/2018 | dBs | 88 | 4,180 | 48 |  |  | 27/Apr | 24/May | 06/May |  |  | Fledged | Eggs |
| 8 | M | 08/05/2017 | Ongoing | dBs | 197 | 9,226 | 47 |  |  | 30/Apr | 24/May | 30/Apr | 28/May |  | Fledged | Fledged |
| 9 | F | 08/05/2017 | Ongoing | dBs | 46 | 2,173 | 47 |  |  | 30/Apr | 26/May |  |  |  | Died | No nest |
| 10 | F | 10/05/2017 | Ongoing | dBs | 146 | 6,679 | 46 |  |  | 27/Apr | 25/May | 16/May | 07/Jun |  | Died | Fledged |
| 11 | M | 12/05/2017 | Ongoing | dBs | 120 | 5,627 | 47 |  |  | 26/Apr |  | 30/Apr | 29/May |  | Eggs | Fledged |
| 12 | F | 19/05/2017 | Ongoing | dBs | 184 | 8,531 | 46 |  |  | 09/May | 31/May | 15/May | 05/Jun |  | Fledged | Fledged |
|  |  |  | **Mean** |  | **128** | **6,021** | **47** | **05/May** | **02/Jun** | **03/May** | **28/May** | **10/May** | **04/Jun** |  | |  |
|  |  |  | **Min** |  | **19** | **888** | **46** | **15/Apr** | **20/May** | **26/Apr** | **24/May** | **30/Apr** | **28/May** |  |  |  |
|  |  |  | **Max** |  | **299** | **14,058** | **48** | **02/Jun** | **22/Jun** | **22/May** | **13/Jun** | **22/May** | **13/Jun** |  |  |  |

**Supplementary Table S2:** Overview of habitat types quantified in this study. Initially 31 habitats were taken from the Corine Land Cover database, 5 habitats from different governmental databases and 11 habitats from an extra layer. These 47 layers were reclassified into 7 main habitat categories for the final habitat map: built-up areas, industrial areas, waste processing areas, city green areas, rural green areas and water areas. The first four are together referred to as suburban and urban areas.

| Nr | CLC | Databases | Extra layer | Main habitat categories |
| --- | --- | --- | --- | --- |
| 1 | Continuous urban fabric |  |  | Built-up areas |
| 2 | Discontinuous urban fabric |  |  | Built-up areas |
| 3 | Industrial or commercial units |  |  | Industrial areas |
| 4 | Road and rail networks and associated land |  |  | Industrial areas |
| 5 | Port areas |  |  | Industrial areas |
| 6 | Airports |  |  | Industrial areas |
| 7 | Mineral extraction sites |  |  | Waste processing area |
| 8 | Dump sites |  |  | Waste processing area |
| 9 | Construction sites |  |  | Industrial areas |
| 10 | Green urban areas |  |  | City green areas |
| 11 | Sport and leisure facilities |  |  | City green areas |
| 12 | Non-irrigated arable land |  |  | Rural green areas |
| 13 | Fruit trees and berry plantations |  |  | Rural green areas |
| 14 | Pastures |  |  | Rural green areas |
| 15 | Complex cultivation patterns |  |  | Rural green areas |
| 16 | Land principally occupied by agriculture |  |  | Rural green areas |
| 17 | Broad-leaved forest |  |  | Rural green areas |
| 18 | Coniferous forest |  |  | Rural green areas |
| 19 | Mixed forest |  |  | Rural green areas |
| 20 | Natural grasslands |  |  | Rural green areas |
| 21 | Moors and heathland |  |  | Rural green areas |
| 22 | Transitional woodland-shrub |  |  | Rural green areas |
| 23 | Beaches, dunes, sands |  |  | Rural green areas |
| 24 | Inland marshes |  |  | Rural green areas |
| 25 | Peat bogs |  |  | Rural green areas |
| 26 | Salt marshes |  |  | Water areas |
| 27 | Intertidal flats |  |  | Water areas |
| 28 | Water courses |  |  | Water areas |
| 29 | Water bodies |  |  | Water areas |
| 30 | Estuaries |  |  | Water areas |
| 31 | Sea and ocean |  |  | Water areas |
| 32 |  | Landfills |  | Waste processing area |
| 33 |  | Green Spaces |  | City green areas |
| 34 |  | Allotment |  | City green areas |
| 35 |  | Rivers Bristol |  | Water areas |
| 36 |  | Estuary |  | Water areas |
| 37 |  |  | Shortwood Landfill | Waste processing area |
| 38 |  |  | Northway Landfill | Waste processing area |
| 39 |  |  | Sewage Works | Waste processing area |
| 40 |  |  | Lower Compton | Waste processing area |
| 41 |  |  | Bristol Waste Com | Waste processing area |
| 42 |  |  | Other landfills | Waste processing area |
| 43 |  |  | Sport fields | City green areas |
| 44 |  |  | Golf courses | City green areas |
| 45 |  |  | Avon River | Water areas |
| 46 |  |  | Nest dBs | Built-up areas |
| 47 |  |  | Nest ASSL | Built-up areas |

**Supplementary Table S3:** Definition of 10 activity classes of Lesser black-backed gulls, used to annotate tri-axial acceleration data. These activity classes were combined to create 7 final activity classes: soaring, flapping, extreme flapping (exflap), mixed flight (mixed), stationary, walking and other. Modified from Shamoun-Baranes *et al.*^59^.

| **General activity** | **Activity class** | **Final class** | **Description** |
| --- | --- | --- | --- |
| Flight | Soaring | Flight | Flight with no wing beat, includes climbing and gliding |
|  | Flapping | Flight | Flapping flight with regular wing beat |
|  | ExFlap | Flight | Irregular and intense wing beat, e.g. during take-off |
|  | Mixed | Flight | Mixed flap-gliding |
| Stationary | Stationary | Stationary | Sitting or standing on land or static structure at sea |
|  | Boat | Stationary | Sitting or standing on a boat |
| Terrestrial locomotion | Walking | Walking | Walking |
|  | Pecking | Walking | Walk and peck. |
| Float | Float | Other | Floating with the currents at sea |
| Other | Other | Other | Activity signal that doesn't fit in the above classes. |

**Supplementary dataset 1**

Dataset used for model 1. The variables are explained as follows:

- id identification of individuals (2-12)
- year year when GPS fix was taken (2016)
- idYear combination of individual and year (2_2016)
- habitat habitat where the GPS fix was taken (see Table S2)
- breedingPhase breeding phase when the GPS fix was taken (see methods, paper)
- duration duration of time spent (in hours)
- totalDuration total time spent per 2-week interval (in hours)

**Supplementary dataset 2**

Dataset used for model 2. The variables are explained as follows:

- id identification of individuals (2-12)
- year year when GPS fix was taken (2016)
- idYear combination of individual and year (2_2016)
- behaviour behaviour of individual when GPS fix was taken (see Table S3)
- habitat habitat where the GPS fix was taken (see Table S2)
- breedingPhase breeding phase when the GPS fix was taken (see methods, paper)
- duration duration of time spent (in hours)
- totalDuration total time spent per 2-week interval (in hours)

**Supplementary dataset 3**

The raw dataset used to create datasets 1-2. The variables are explained as follows:

- id identification of individuals (2-12)
- date date and time when GPS fix was taken (yyyy/mm/dd hh:mm:ss)
- latitude latitude in decimal degrees (xx.xxxxxxx)
- longitude longitude in decimal degrees (xx.xxxxxxx)
- altitude altitude above ground level (in meters)
- behaviour behaviour of individual when GPS fix was taken (see Table S3)
- breedingSeason breeding season when the GPS fix was taken (2016,2017,2018)
- breedingPhase breeding phase when the GPS fix was taken (see methods, paper)
- breedingStage breeding stage when the GPS fix was taken
- habitat habitat where the GPS fix was taken (see Table S2)
- duration duration between subsequent GPS fixes (in secs) after 30-min filter
